# 

**Published:** 1894-06

**Authors:** 


### Society Notes.

**The Twenty-Seventh Quarterly Meeting** of the Austin District Medical Society will be held in the Knights of Pythias hall, Austin, Texas, Thursday, June 21, 1894.

PROGRAMME.

1. "After Treatment of Abortions," by Dr. J. Cummings; discussion opened by Dr. J. C. Anderson and Dr. T. D. Wooten.
2. "The Cause and Prevention of Prostitution," by Dr. Isaac J. Jones; discussion opened by Dr. B. M. Worsham and Dr. J. W. Carhart.

"The Modern treatment of Hemorrhoids," by Dr. T. J. Bennett; discussion opened by Dr. J. S. Brown and Dr. A. Garwood.

4. "Spinal Concussion," by Dr. R. M. Swearingen; discussion opened by Dr. F. R. Martin and Dr. F. S. White.
5. Voluntary papers and reports of cases.

T. J. BENNETT, President.

S. E. HUDSON, Secretary.

---

**Central Texas Medical Association** will hold its semi-annual session in Waco, July 10th and 11th prox. Dr. J. M. Frazier, late of Morgan, now of Belton, is President, and Dr. H. S. Taylor, Secretary.

Dr. O. I. Halbert, the county physician, will have a paper on Tuberculosis.

Dr. W. R. Blailock, a paper on Asepsis and Antisepsis; Dr. R.

### INDEX TO VOL. IX.

Absorption of fibroid tumors of the uterus ................ 130
Anæsthesia, statistics relative to ........................ 398
Antidotal powers (alleged) of permanganate of potassium ..... 509
Alimentation of infants ........................ ...... 513
Abscess of the liver, Zancaral ..... . .................... 340
Acute alcoholism, hot water in ................... ........ 391
"Amick" ............ .... ............................ 135
Atypical forms of disease, Stroud ................ ...... 62
An unworthy suspicion ........... .... ............... 410
A case of drowning, Osborn .............................. 373
Amick's press dispatches ................................ 294
A literary bomb-shell ................................... 459
A mare's nest ... ....................................... 461
An improved method of brain localization in epilepsy, Hadra ... 455
Antipyretic, the new .................................... 462
A libel on the profession ............. ................ 596
A merited honor ......................................... 604
Autopsy, an interesting ..... ........................... 132
Atlas of rare skin diseases ......... ................... 340
Antipyretics .............. ............................ 618
Bill, a, to establish a department of public health ....... .... 474
Bills now in Congress, the two ........... .............. 464
Banquet, that ................................ ... 602
Biographical, McLaughlin ........ ...................... 605
Broad ligament, ligature of ............................. 28
Breech presentations .................................... 50
Book Notices and Reviews—
Reactions, Fluckiger ............................ 544
Medical Diagnosis, a clinical text-book of, A. Vierordt .. 544
Hernia, T. H. Manley ............................ 546
Transactions Southern S. and G. Ass'n .............. 546
Materia medica and therapeutics, treatise on, Bartholow. 547
Practice of surgery, lectures on, Senn ............... 547
Cholera, a chapter on, Walter Vought ............... 547
Eye, surg. anatomy and surgery of the, Tuttle ........ 548
Legal medicine, a system of, A. McL. Hamilton ....... 548
Surgery, a practical treatise on, M. Moullin ........... 578
Reference hand-book med. science, supplement to, Buck. 580
Annual universal medical science, Sajous ............. 581
Theory and practice of medicine, text-book on, Pepper, 92, 582
Bacteriology, essentials of, M. V. Ball ................ 583
Operation blank, Keen ............................ 583
Sterility in women, De Sinety ...................... 584
Massage, recent developments in, Graham .......... .... 584
Gynecology, modern, Bushong ...................... 420
Theory and practice of medicine, Whittaker .......... 422

Pharmacy, principles and practiçe of, Kennedy & Morris 422
Naphey's modern therapeutics, Smith ................ 422
Rectum and anus, diseases of, Kelsey ............... 425
Medical science, a dictionary of, Dunglison .......... 362
Medicine, a text-book of, Strumpel .................. 363
Neurasthenia, electro-therapeutics of, Robinson ....... 364
Bacterial poisons, Gamaleia ....................... 364
Minor surgery, essentials of, Edw. Martin ............ 307
Pocket day-book, Leonard's ...................... 307
Insanity, hand-book of. Kirchhoff .................. 245
Materia medica and therapeutics, Shoemaker .......... 247
Appendicitis and perityphlitis, Tallamon ............. 248
International clinics, Keating and others ............ 248
Gynecology, American text-book of, Kelsey, Pryor, Bedford and others ........................... 249
Genito-urinary diseases, syphilology and dermatology, a system of, Morrow ........................ 197
Brain surgery, Allen Starr ........................ 189
Local therapeutics, a hand-book of ................. 196
Inebriety, diseases of, Caruthers ................... 191
Medical consultation book, Hachenberg .............. 192
Dunglison's new pronouncing Dictionary ............ 192
Nervous system, diseases of, Hirt ................. 90
Practical diagnosis, lessons in, Loomis .............. 93
Psychopathia sexualis, Kroft Ebing .................. 41
Human Physiology, elements of, Sterling ............ 42
Cholera, Archie Stockwell ....................... 42
Dissection, guide to, Hayes ...................... 43
Agnew, life of Dr. D. Hayes, T. H. Adams ........... 44
Treatment, a year book of (Lea Bros. Pub.) .......... 45
Fermentation, Infection and Immunity, McLaughlin .... 45
Diseases of the skin, Pye. Smith ................... 76
Atlas of rare skin diseases ........................ 341
Prison life, etc. .............................. 647
Chioroform in labor, Dunagan ......................... 119
Castration of sexual perverts, Daniel ..................... 255
Christmas Greeting ................................ 291
Compound fracture lower extremities, Menger .............. 216
Cæsarian section .................................. 131
Creasote in latent tuberculosis ........................ 339
Cocaine wakefulness ............................... 396
Catarthinic acid, the uses of .......................... 450
Conium in torticollis ............................... 508
Correspondence—
Secretary West and the buffet banquet, Wallace ...... 78
Some cranial surgery, Pugh ...................... 170
Hammond, Dr. W. A., letter from .................. 225
Diagnosis wanted, Barham ....................... 226
Congenital anchylosis of fingers, Coffey ............. 227
Carbuncles, treatment of by injections of carbolic acid ...... 276
Caffein in alcoholism ................................ 24

Congenital anchylosis of sixteen joints, Milliken.............. 150
Country doctor, the.................................. 184
Commencement day ..................................... 600
Cysts of the spermatic cord and testicle, Thompson.......... 433
Conservative gynecology ............................... 405
Confederates all right.................................. 411
Code of ethics, the, DeCausey.... ................ ....... 328
Cervical dysmenorrhœa.................................. 29
Creasote in phthisis pulmonalis.... ...................... 277
Cystitis, ammonium chloride in............................ 280
Chloroform as a tænicide................................ 281
Chlorosis, treatment of.................... ........... 282
Cardiac diseases, large doses of strychnia in................ 283
Cyst (papillary) of the paro-ophoron...................... 184
Diabetes coma, recovery from............................. 517
Disputed points in surgery of pelvic disease................ 512
Deaths from lead poisoning due to diachylon plaster.......... 68
Diphtheria treated by tartaric acid and corrosive sublimate..... 68
Diphtheria, treatment of........................... ..... 515
Diseases of the skin, Pye Smith on .......... ............ 76
Diaza-Reaction, the, of Ehrlich........................... 389
Disinfection in an epidemic of pneumonia ................ 392
Dislocation of semiluna cartilege of knee................... 400
Drowning, a case of, Osborn.............................. 373
Diagnosis of diphtheria.................................. 298
Dysentery, a Mexican remedy for.......................... 211
Department public health, a.... ............ ........... 474
Do newspapers foster abortion? Taylor.................... 446
Dislocation (outward) of knee, report of case, Bennett ...... 505
Death from use of pentol ................................ 281
Department of Therapeutics .............................. 634
Department of Practice of Medicine....... ............... 639
Enteroplexy ........................... ............. 221
Elimination of mercury.... .............................. 76
Ergot in labor.......................................... 448
Erysipelas, treatment of, with hypodermic injections of carbolic acid.......................... ............... 518
Ex-Confederates disfranchised............................ 347
Electricity in medicine, Morris........................... 99
Ethical relationship of the medical profession to life insurance companies, Stout ................................ ..... 493
Ether anæsthesia, the discovery of.................... .. 530
Elongation of the cervix as cause of obstructed labor........ 29
Ectopic gestation, etiology of............ .............. 289
Facial erysipelas, nitrate of aconitine in................... 450
Female bladder, direct examination of.... ... ........... 511
First (the) medical organization in the U. S................. 277
Function of the pharmacist............................... 83
Guiacol, external uses of................................ 570
Galinol in psoriasis and eczema ............ ........... 13
Gallanol ............................................... 169

Graves' disease, prthology and treatment of.................. 70
Gunshot wounds, treatment of.......................... 401
Guiacol as an antipyretic............................. 451
Gastro Hysteropexy as a safe and reliable means of correcting prolapsus and retro-displacements of the uterus.......... 618
Hydrocephalus......................................... 221
Hysterectomy vs. High Amputation ..................... 288
Hæmorrhoids, operative treatment of................... 221
Hysterectomy.......................................... 334
Hysterectomy, vaginal ................................ 335
Hydrosalpinx prolapsed into Douglas' pouch ........... 630
Intestinal obstruction by biliary calculi............. 400
Infantile scorbutus .................................. 517
Idiopathic peritonitis ............................... 518
Incarcerated hernia, belladonna in ................... 25
Insurance and assurance............................... 292
Intelligent treatment of domestic animals ............ 81
In memoriam, Dr. Wagley .............................. 358
Indecent publications in newspapers................... 466
Journal, the, and the Association .................... 527
Kola-nut, therapeutic applications of ................ 72
Leprosy, Dyer......................................... 558
Law vs. justice, Kennedy. ............................ 565
Libel (a) on the profession........................... 596
Lysol in obstetric practice .......................... 508
Medical News and Mis., 87, 606, 231, 359, 413, 540, 468, 139, 299, 644
Mexican remedy for dysentery, a, Knox................. 211
Mexican sanitation. Sanitation on the Rio Grande...... 231
Mastiod operation, the, Church........................ 503
Magnetic healers and sight diagnosticians............. 635
McLaughlin, Dr. J. W., biography of................... 605
Medical department University of Texas................ 593
Necrological..........................................347–353
Death of Dr. Wagley, J. L............................. 347
" " " Paine, C. F..................................... 348
" " " de Steiger....................................... 350
" " " Waters ......................................... 353
Night sweats, treatment of............................ 339
Neurosis following enteric fever...................... 390
Otitis media, R. E. Moss.............................. 271
Ostitis, M. M. Smith.................................. 437
Opium disease in infants and children................. 478
Outward dislocation of knee, report of a case, Bennett. ....... 505
Our esteemed contemporary, H. H. Harralson............ 182
Our "Wiley" friend ................................... 176
Plural births, hereditary tendency to ................ 131
Preventive Inoculations against hydrophobia .......... 388
Pelvic abscess with gangrene of vermiform process, Schenck ... 113
Puerperal eclampsia, observations on, Cunningham...... 320
Pelvic abscess........................................ 287
Pyorrhœa alveolaris, Caspar........................... 470
Puerperal sepsis...................................... 477

Polio-myelitis, Eskridge .......... 160
Phenocele, hydro chloride of .......... 23
Psoriasis, treatment of .......... 25
Pental, death from use of .......... 281
Parasitic entozoa encountered in Texas .......... 613
Pot-Pourri .......... 640
Quinine as an oxytocic .......... 339
Rupture of the uterus .......... 130
Rest vs. exercise in the treatment of pulmony consumption .......... 71
Railway spine, a case of, A. J. Smith .......... 51
Report of a case of the difficulties in the primipara .......... 57
Removal of an ovarian tumor, Keen .......... 64
Remedy for dysentery, a Mexican .......... 211
Report of a case of outward dislocation of knee .......... 505
Railway surgeon, the, and the law, Clark Bell .......... 553
Shaking palsy, Smith .......... 126
Symphysiotomy (combined) and craniotomy in osteomalacial pelvis 129
Strangulated obturator hernia of ovary .......... 131
Stump, treatment of by supravaginal hysterectomy .......... 337
Skin affections, Brown—Sequard's fluid in .......... 396
Syphilis, sulphate of copper in treatment of .......... 447
Spurious pragnancy, McVey .......... 60
Some of the hardships of a physician, Tally .......... 378
Specific remedies in infectious disease .......... 385
Straining at a gnat .......... 297
Some observations on Thiersch's method of skin grafting .......... 498
Syphilitic fever .......... 26
Suppurating peri-uterine lesions, 59 consecutive operations for .......... 285
Softness of fetal cranium—tardy labor due to .......... 286
Society notes—
- Austin district medical society .......... 134, 290, 343, 349, 641
- Johnson county medical society .......... 132
- S. E. Texas medical society .......... 134
- Pan-American medical congress .......... 80
- S. W. Texas medical society .......... 289
- Galveston county medical society .......... 290, 343
- Central Texas medical society .......... 346
- Terrell medical society .......... 401
- International medical congress .......... 402
- Texas state medical association .......... 419, 452, 525, 517
- Texas state pharmacy association .......... 592

Sorry bird, the Hot Springs .......... 153
Section R. R. surgery medico-legal society .......... 359
Syphilitic epididymitis .......... 616
The deadly doctor .......... 643
Tube cular peritonitis, a new method in .......... 220
Tuberculosis in the negro .......... 70
Thilanin, the uses of .......... 42
Thioform .......... 338
Therapeutic uses of exalgin .......... 394
Treatment of cholera, the, by hypodermoclysis and enteroclysis .......... 121
Treatment, nasal, in ear troubles .......... 74

Treatment of night sweats .......... 339
Treatment of uterine fibroids, Goellett .......... 444
" of phthisis .......... 390
" of diphtheria .......... 515
" of tetanus .......... 393
" of epilepsy .......... 395
" of convulsive attacks by anesthetics .......... 395
" of typhoid fever by thymic acid .......... 121
" for tuberculosis, a new .......... 125
" of acute pneumonia, ice in .......... 125
" of pleurisy, salicylates in .......... 128
" of hæmoptysis .......... 129
" of ephilides by ecorchement .......... 75
Tolerance of nitro glycerine .......... 73
Tuberculin treatment in Egypt .......... 397
Typhoid fever, boric acid in .......... 397
Transplantation of cutaneous flaps .......... 399
" of free cutaneous flaps .......... 399
Typhlitis, medicinal treatment of .......... 448
Thyroid feeding in skin disease .......... 169
Typhoid fever, diagnosis of .......... 278
The outlook of the profession, Payne .......... 67
Travesty on religion and law .......... 412
Tuberculosis, the French congress on, Bibb .......... 199
Tongue traction, Shearer .......... 219
Texas delegates to Pan-American medical congress .......... 86
Traumatism as a factor in ovarian pathology, Kennedy .......... 440
The new antipyretic .......... 472
The JOURNAL and the association .......... 527
Texas medical law, the .......... 537
That decision .......... 540
Tubercular meningitis, Smith .......... 147
Teaching anatomy in medical schools, Flavin .......... 155
The Texas medical college, third session .......... 178
The doctor's lament .......... 183
Ulcer of the foot, Jackson .......... 68
Uterine hemorrhage, discussion on .......... 344
" " Keiller .......... 313
" fibroids, treatment of, Goellett .......... 446
Umbilical hemorrhage, Grayson .......... 507
United confederate veterans .......... 538
Ulcers, the tin-plate treatment of .......... 24
Uterine cancer, early diagnosis of .......... 29
Varicocele and its radical cure, Milliken .......... 332
What a fish a frog is .......... 229
Was it hydrophobia? Brown & Baily .......... 502
Whited sepulchre, a .......... 539
What's in a name? .......... 173
Warts and excrescences .......... 169
Yellow fever .......... 126
Yellow fever from a medico-geographical and prophylactic standpoint .......... 279

